# Efficacy of statins on renal function in patients with chronic kidney disease: a systematic review and meta-analysis

**DOI:** 10.1080/0886022X.2021.1915799

**Published:** 2021-04-29

**Authors:** Lin Zhao, Shu Li, Ying Gao

**Affiliations:** a International Medical School, Tianjin Medical University, Tianjin, P.R. China; b School of Public Health, Tianjin Medical University, Tianjin, P.R. China; c Health Management Center, Tianjin Medical University General Hospital, Tianjin, P.R. China

**Keywords:** Statins, chronic kidney disease, renal function, meta-analysis

## Abstract

**Background:**

Studies have shown that the use of statins could significantly improve lipid profiles; however, it remains controversial whether the use of statins could improve renal function in patients with chronic kidney disease (CKD). Therefore, we conducted a meta-analysis of randomized controlled trials (RCTs) to evaluate the effects of statins on renal function in patients with CKD.

**Methods:**

We systematically searched PubMed, EMBASE, and the Cochrane Library databases for eligible RCTs from inception to October 2020. Pooled effect estimates were assigned as weighted mean differences (WMDs) with 95% confidence intervals (CIs) using the random-effects model.

**Results:**

We selected 33 RCTs that recruited 37,391 patients with CKD patients. The summary results suggested that statin use significantly reduced urinary albumin (WMD: −2.04; 95%CI: −3.53 to −0.56; *p* = .007) and protein (WMD: −0.58; 95%CI: −0.95 to −0.21; *p* = .002) excretions and increased creatinine clearance (WMD: 0.86; 95%CI: 0.32–1.41; *p* = .002). However, there were no significant differences between statin and control groups in terms of changes in estimated glomerular filtration rate (WMD: 0.38; 95%CI: −0.04 to 0.79; *p* = .075), and serum creatinine levels (WMD: −0.07; 95%CI: −0.25, 0.12; *p* = .475).

**Conclusions:**

We found that statin use in patients with CKD may slow CKD progression by lowering urinary albumin and protein excretions or increasing creatinine clearance. Further large-scale RCTs should be conducted to evaluate the long-term effects of statins on renal outcomes. **Abbreviations:** CKD: chronic kidney disease; RCT: randomized controlled trials; WMD: weighted mean differences; CI: confidence intervals; ACEI: angiotensin-converting enzyme inhibitors; eGFR: estimated glomerular filtration rate

## Introduction

Chronic kidney disease (CKD) is a common disorder that can cause cardiovascular disease, kidney failure, and other complications. CKD, with an increasing prevalence, affects more than 10% of the global population [1]. In the United States of America (USA), an estimated 9.6% of non-institutionalized adults suffer from CKD [2,3]. Studies conducted in Europe, Australia, and Asia confirm the high prevalence of CKD [4–6]. However, the prevalence of CKD in many developing countries remains unknown [7]. CKD has many potential causes that vary in frequency among populations [8]. Renovascular disease is one of the risk factors for developing CKD and worsening renal outcomes [9]. Some proposed mechanisms for progressive CKD in patients with renovascular disease include endothelial dysfunction, oxidative stress, and systemic inflammation of the glomerular capillary wall [10]. Renal replacement therapy is currently the most effective CKD treatment modality; however, the administration of renal replacement therapy in low- and middle-income countries is limited because it is not readily available in these countries. Further, most patients die of kidney failure without receiving dialysis or transplantation [11]. Thus, it is necessary to find alternative strategies to improve the prognosis of CKD.

Lipid-lowering therapies may improve renal function and lower albuminuria as reported in some previous meta-analyses [12,13]. They found that lipid reduction could improve renal function and decrease proteinuria in patients with CKD [13]. Although the abovementioned meta-analyses reported the beneficial effects of statins on pathologic albuminuria, larger studies are required to assess the validity of these findings and determine if statins can also reduce cardiovascular or end-stage renal disease occurrence [12]. Another study found that a combined approach using angiotensin-converting enzyme inhibitors (ACEI) and statins could represent a therapeutic option for patients with advanced renal disease in whom ACEI alone failed to substantially reduce proteinuria and renal injury [14]. Some scholars believe that this may be due to the anti-inflammatory effect of statins and improved endothelial function [14]. Previous meta-analyses that evaluated the effect of statins on renal outcomes did not focus on patients with CKD [15]. Therefore, we conducted this meta-analysis, based on randomized controlled trials (RCTs), to evaluate the effects of statins on renal function in patients with CKD.

## Methods

### Search strategy and eligibility criteria

According to the transparent reporting of systematic reviews and meta-analyses 2009 (PRISMA checklist) [16], two reviewers independently identified relevant studies in the PubMed, EMBASE, and Cochrane library databases from inception to October 2020. This review included only studies published in English, and the following terms were applied in our search: CKD, chronic renal disease, chronic nephropathy, statin, atorvastatin, simvastatin, rosuvastatin, pravastatin, lovastatin, fluvastatin, cerivastatin, mevastatin, pitavastatin, dyslipidemia, hyperlipidemia, hypercholesterolemia, hyperlipoproteinemia, hypertriglyceridemia, human, and RCTs. The details of the search strategies for each database are shown in Supplemental 1. If a dataset was published multiple times, the most relevant publication was included, and the others supplemented it.

Studies were included if they met the following criteria: (1) study design: RCT; (2) patients: CKD; (3) intervention: statins, irrespective of dose and types; (4) control: usual care, placebo, dietary therapy, or low dose statins (less than half of the dose in intervention group); and (5) outcomes: the study had to reported at least 1 of following outcomes: (estimated glomerular filtration rate [eGFR] 186 × [serum creatinine, mg/dL]^−1.154^ × [age, years]^−0.203^ × [0.742 if female] × [1.210 if black]), urinary albumin excretion, creatinine clearance ([140-age]×body weight [kg]/[814.5 × serum creatinine [umol/L] × 1000 × 0.85 if female]), serum creatinine (Jaffe Kinetic method), and urinary protein excretion. Reviews, case reports, letters, mechanism studies, and non-human studies were excluded. After an initial screening of the study titles and abstracts, the full texts of potentially eligible studies were read to assess whether the study could be included in the meta-analysis. This process was performed by two reviewers (SL and YG), and inconsistent results between reviewers were settled by group discussion until a consensus was reached.

### Data extraction and quality assessment

Data were independently extracted from the selected studies by two reviewers (SL and YG) using standardized criteria. The following items were extracted: first author’s surname, year of publication, country, number of participants, mean age, and baseline eGFR in the intervention and control groups, intervention, control, follow-up duration, and reported outcomes. Quality assessment was performed simultaneously by two reviewers (SL and YG) using the Jadad scale (with scores ranging from 0 to 5), and based on randomization, blinding, allocation concealment, withdrawals and dropouts, and the use of intention-to-treat analysis [17]. Any conflicts between reviewers for data abstraction and quality assessment were settled by a third reviewer (LZ), who reviewed the full-text of retrieved studies.

### Statistical analysis

The treatment efficacy of statins on renal function improvement in patients with CKD was assigned as continuous data, and the pooled weighted mean differences (WMDs) with 95% confidence intervals (CIs) were calculated using the random-effects model, which considering the varies underlying included studies [18,19]. The heterogeneity among included studies was assessed using the *I*^2^ statistic and Q statistic [20,21]. Sensitivity analysis was conducted by leave-one-out to assess the robustness of a pooled conclusion [22]. Subgroup analysis was conducted according to year of publication (before 2010, 2010, or after), country (Asia, other), sample size (≥100, <100), mean age (≥65.0, <65.0 years), statin type (atorvastatin, fluvastatin, pitavastatin, pravastatin, rosuvastatin, and simvastatin), follow-up duration (≥12.0, <12.0 months), and study quality (high [Jadad score 4 or 5], low [Jadad score 0–3]); the difference between subgroups was assessed using the interaction *p* test [23]. The funnel plot, Egger, and Begg test results were used to assess potential publication bias [24,25]. The inspection level was 2-sided, and statistical significance was set at *p*<.05. All statistical analyses were performed using STATA software version 10.0 (StataCorp, College Station, TX).

## Results

### Literature search

**Figure 1.**
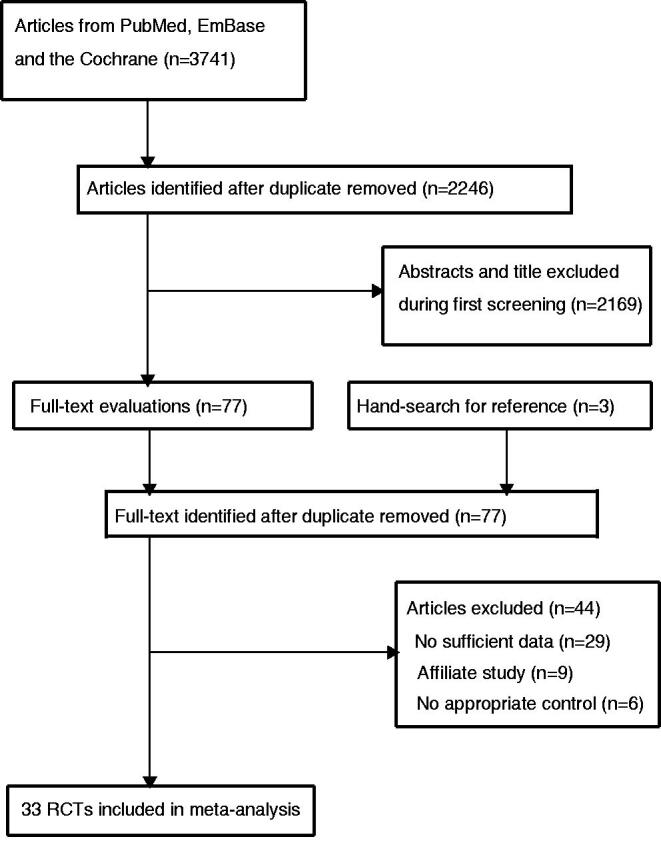
Flow chart of the study selection process.

A total of 3741 published studies were initially identified, of which 2246 potentially relevant studies were retained after duplicates were removed. Subsequently, 2169 were excluded owing to irrelevant topics. The remaining 77 studies were retrieved for further full-text evaluations, and 33 RCTs met the inclusion criteria [26–58]. By reviewing the reference lists of these studies, we found three potentially eligible studies, and these studies were contained in initial electronic searches. Details of the study selection process are shown in Figure 1.

### Study characteristics

We included 33 RCTs involving 37,391 patients with CKD; the baseline characteristics of included studies are summarized in Table 1. The duration of follow-up ranged from 10 d to 72.0 months and 28 to 16,245 patients were included in each individual trial. The intervention strategies included the administration of atorvastatin, cerivastatin, fluvastatin, pitavastatin, pravastatin, rosuvastatin, and simvastatin. Study quality was assessed using the Jadad scale; 3, 10, 13, and 7 trials scored 5, 4, 3, and 2, respectively.

**Table 1.** Information extracted from the studies included in the meta-analysis.

Study	Country	Sample size (intervention/control)	Mean age (years) (Intervention/control)	Baseline eGFR (mL/min/ 1.73 m^2^) (intervention/ control)	Intervention	Control	Follow-up (months)	Study quality
Nielsen et al. [26]	UK	8/10	65/65	96.6/97.1	Simvastatin (10 mg/d)	Placebo	9	3
Tonolo et al. [27]	Italy	10/9	60/62	NA	Simvastatin (20 mg/d)	Placebo	12	2
Imai et al. [28]	Japan	32/25	58.5/49.5	NA	Pravastatin (5, 10 mg/d)	Placebo	6	3
Santos et al. [29]	Brazil	34/33	44.3/42.2	NA	Simvastatin (10 mg/d)	Placebo	6	3
Nakamura et al. [30]	Japan	30/30	58/55	NA	Cerivastatin (0.15 mg/d)	Placebo	6	3
Gheith et al. [31]	Egypt	21/22	23/22.2	NA	Fluvastatin (20 mg/d)	Usual care	12	2
Nakamura et al. [32]	Japan	20/20	40.8/38.8	NA	Cerivastatin (0.15 mg/d)	Placebo	6	3
Bianchi et al. [33]	US	28/28	56.5/56.8	NA	Atorvastatin (40 mg/d)	Usual care	12	3
Fellstrom et al. [34]	Switzerland	1050/1052	49.5/50.0	52.9/52.1	Fluvastatin (40 and 80 mg/d)	Placebo	72	4
Yasuda et al. [35]	Japan	39/41	57/58	59.0/60.0	Fluvastatin (20 mg/d)	Dietary therapy	12	2
Asselbergs et al. [36]	The Netherlands	433/431	52.1/50.5	NA	Pravastatin (40 mg/d	Placebo	46	4
Tonelli et al. [37]	Canada	1702/1700	63.1/63.5	52.7/52.7	Pravastatin (40 mg/d)	Placebo	60	4
		6479/6364	57.5/57.5	73.8/73.8				
Nakamura et al. [38]	Japan	10/10	51/49	NA	Pitavastatin (1 mg/d)	Usual care	12	2
Atthobari et al. [39]	The Netherlands	400/388	52.1/50.9	75.7/75.5	Pravastatin (40 mg/d)	Placebo	48	4
Goicoechea et al. [40]	Spain	44/19	66.2/70.0	42.8/44.2	Atorvastatin (20 mg/d)	Usual care	6	2
Nakamura et al. [41]	Japan	15/15	39.5/40.5	NA	Pitavastatin (1 mg/d)	Placebo	6	4
Nanayakkara et al. [42]	The Netherlands	47/46	54.0/52.0	32.0/35.0	Pravastatin (40 mg/d)	Placebo	24	5
Rahman et al. [43]	US, Puerto Rico, US Virgin Islands, and Canada	779/778	66.7/66.6	51.5/51.0	Pravastatin (40 mg/d)	Usual care	57.6	4
		2903/2960	67.0/67.0	75.4/75.2				
Sawara et al. [44]	Japan	22/16	63.8/67.0	50.7/57.3	Rosuvastatin (2.5 mg/dL)	Usual care	12	2
Colhoun et al. [45]	UK	482/488	65.0/65.0	53.5/54.1	Atorvastatin (10 mg/d)	Placebo	46.8	5
Koren et al. [46]	US	286/293	65.6/64.8	51.3/51.1	Atorvastatin (<80 mg/d)	Usual care	54.3	3
Fassett et al. [47]	Australia	58/65	60.0/60.3	31.9/29.1	Atorvastatin (10 mg/d)	Placebo	30	4
Fassett et al. [48]	Australia	29/20	53.0/49.0	58.5/49.9	Pravastatin (20 mg/d)	Usual care	24	3
Ruggenenti et al. [49]	Italy	87/93	51.4/51.4	56.2/52.5	Fluvastatin (40 mg/d)	Usual care	6	3
Abe et al. [50]	Japan	52/52	64.5/64.9	70.4/69.3	Rosuvastatin (<10 mg/d)	Usual care	6	3
Fassett et al. [51]	Australia	56/61	59.6/60.2	32.0/29.2	Rosuvastatin (10 mg/d)	Placebo	30	4
Haynes et al. [52]	Europe	3116/3129	63.0/63.0	26.6/26.6	Simvastatin (20 mg/d)	Placebo	57.6	4
Zeeuw et al. [53]	Argentina, Brazil, Bulgaria, Canada, Denmark, France, Hungary, Italy, Mexico, Romania, and the US	116/107	56.8/58.5	72.6/68.8	Rosuvastatin (40 mg/d)	Rosuvastatin 10 mg	12	5
Takazakura et al. [54]	Japan	63/43	62.0/63.0	65.3/61.4	Atorvastatin (10 mg/d) or pravastatin (10 mg/d)	Dietary therapy	12	3
Ohsawa [55]	Japan	14/14	60.6/63.9	48.6/50.1	Pitavastatin (<4 mg/d)	Dietary therapy	12	3
Shehata et al. [56]	Egypt	65/65	55.0/57.0	48.0/49.0	Atorvastatin (80 mg/d)	Placebo	0.3	3
Yazbek et al. [57]	Brazil	51/49	41.2/41.0	NA	Rosuvastatin (10 mg/d) or atorvastatin (10 mg/d)	Usual care	12	2
Kimura et al. [58]	Japan	168/166	63.2/63.1	56.0/54.0	Atorvastatin (5–20 mg/d)	Dietary therapy	24	4

eGFR: estimated glomerular filtration rate.

### Estimated glomerular filtration rate

**Figure 2.**
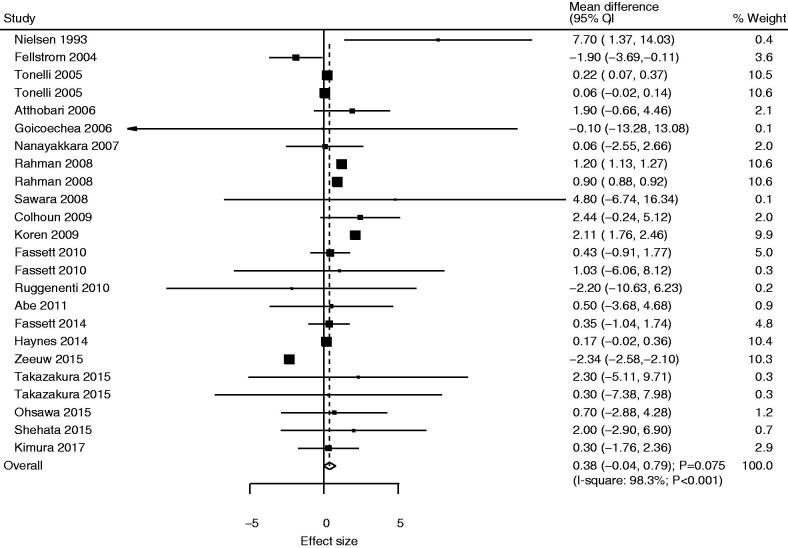
Comparison of estimated glomerular filtration rate (eGFR) change between statin and control groups.

Twenty-one studies reported the effect of statins on eGFR, and the pooled result indicated that the use of statins was not associated with a change in eGFR as compared with the control (WMD: 0.38; 95%CI: −0.04 to 0.79; *p* = .075; Figure 2). Moreover, we observed significant heterogeneity across included trials (*I*^2^=98.3%; *p*< .001). Sensitivity analysis suggested that the pooled conclusion was unstable because of the marginal 95%CI (Supplemental 2). Subgroup analysis revealed that statin use was associated with high eGFR for pooled studies published before 2010, mean patient age ≥65.0 years, atorvastatin use, pravastatin use, and low-quality studies. However, we noted that the use of fluvastatin was associated with a lower eGFR than that in the control group (Table 2). There was no significant publication bias for eGFR (*p* value for Egger: .277; *p* value for Begg: .309; Supplemental 3).

**Table 2.** Subgroup analysis.

Outcomes	Factors	Subgroup	WMD and 95%CI	*p* Value	*I*^2^ (%)	*p* Value for Q statistic	*p* Value between subgroups
eGFR	Year of publication	Before 2010	0.83 (0.44–1.22)	<.001	98.2	<.001	<.001
		2010 or after	0.03 (−1.29 to 1.35)	.966	95.9	<.001	
	Country	Asia	0.57 (−0.98 to 2.13)	.470	0.0	.977	.733
		Other	0.36 (−0.07 to 0.79)	.097	98.7	<.001	
	Sample size	≥100	0.33 (−0.09 to 0.76)	.123	98.9	<.001	.755
		<100	1.13 (−0.65 to 2.90)	.213	0.0	.611	
	Mean age (years)	≥65.0	1.08 (0.78–1.38)	<.001	93.0	<.001	<.001
		<65.0	0.13 (−0.50 to 0.76)	.689	96.8	<.001	
	Statin type	Atorvastatin	1.50 (0.65–2.35)	.001	28.0	.208	<.001
		Fluvastatin	−1.91 (−3.66 to −0.16)	.032	0.0	.946	
		Pitavastatin	0.70 (−2.88 to 4.28)	.702	–	–	
		Pravastatin	0.62 (0.19–1.04)	.004	98.8	<.001	
		Rosuvastatin	−0.60 (−2.89 to 1.70)	.611	82.4	.001	
		Simvastatin	3.24 (−4.01 to 10.50)	.381	81.6	.020	
	Follow-up duration	≥12.0	0.34 (−0.08 to 0.76)	.112	98.7	<.001	.434
		<12.0	1.95 (−1.00 to 4.90)	.196	14.1	.325	
	Study quality	High	0.11 (−0.33 to 0.56)	.615	99.1	<.001	<.001
		Low	2.09 (1.75–2.43)	<.001	0.0	.834	
Urinary albumin excretion rate	Year of publication	Before 2010	−1.65 (−3.20 to −0.11)	.036	99.3	<.001	<.001
		2010 or after	−6.56 (−9.90 to −3.22)	<.001	–	–	
	Country	Asia	−4.92 (−9.83 to 0.00)	.050	96.2	<.001	<.001
		Other	−0.13 (−4.36 to 4.10)	.951	99.5	<.001	
	Sample size	≥100	0.88 (−3.11 to 4.88)	.665	96.2	<.001	<.001
		<100	−3.74 (−5.34 to −2.14)	<.001	99.2	<.001	
	Mean age (years)	≥65.0	−14.60 (−15.74 to −13.46)	<.001	–	–	<.001
		<65.0	0.04 (−1.07 to 1.16)	.938	98.4	<.001	
	Statin type	Cerivastatin	−6.78 (−8.49 to −5.07)	<.001	–	–	<.001
		Fluvastatin	0.32 (−0.32 to 0.96)	.324	97.9	<.001	
		Pitavastatin	−7.46 (−14.18 to −0.74)	.030	–	–	
		Pravastatin	2.81 (0.04–5.57)	.047	95.1	<.001	
		Rosuvastatin	−6.56 (−9.90 to −3.22)	<.001	–	–	
		Simvastatin	−2.41 (−27.47 to 22.65)	.850	95.1	<.001	
	Follow-up duration	≥12.0	1.63 (0.49–2.78)	.005	98.5	<.001	<.001
		<12.0	−9.41 (−15.48 to −3.34)	.002	97.0	<.001	
	Study quality	High	2.81 (0.04–5.57)	.047	95.1	<.001	<.001
		Low	−4.87 (−6.55 to −3.19)	<.001	99.2	<.001	
Creatinine clearance	Year of publication	Before 2010	−0.02 (−0.06 to 0.01)	.185	0.0	.678	<.001
		2010 or after	2.22 (−1.37 to 5.81)	.226	83.5	<.001	
	Country	Asia	−0.30 (-4.96 to 4.36)	.900	76.7	.014	.650
		Other	0.95 (0.39–1.50)	.001	94.9	<.001	
	Sample size	≥100	−0.02 (−0.06 to 0.02)	.292	0.0	.556	.552
		<100	1.71 (−1.09 to 4.52)	.231	94.4	<.001	
	Mean age (years)	≥65.0	–	–	–	–	–
		<65.0	0.86 (0.32–1.41)	.002	92.8	<.001	
	Statin type	Atorvastatin	4.80 (3.90–5.70)	<.001	–	–	<.001
		Cerivastatin	−4.00 (−10.85 to 2.85)	.252	–	–	
		Fluvastatin	1.50 (−9.16 to 12.17)	.782	64.7	.059	
		Pravastatin	1.56 (−2.34 to 5.46)	.433	79.7	.026	
		Rosuvastatin	−0.02 (−0.06 to 0.02)	.293	–	–	
		Simvastatin	3.30 (−1.74 to 8.34)	.199	0.0	.869	
	Follow-up duration	≥12.0	0.82 (0.26–1.37)	.004	95.8	<.001	.153
		<12.0	1.02 (−3.51 to 5.55)	.659	42.5	.157	
	Study quality	High	−0.02 (−0.06 to 0.02)	.293	–	–	.556
		Low	1.48 (−1.25 to 4.21)	.287	93.6	<.001	
Serum creatinine	Year of publication	Before 2010	−0.14 (−0.54 to 0.26)	.504	97.0	<.001	<.001
		2010 or after	−0.01 (−0.06 to 0.05)	.774	3.8	.374	
	Country	Asia	−0.10 (−0.38 to 0.17)	.447	96.8	<.001	.063
		Other	−0.00 (−0.14 to 0.13)	.946	35.4	.212	
	Sample size	≥100	−0.01 (−0.06 to 0.05)	.774	3.8	.374	<.001
		<100	−0.14 (−0.54 to 0.26)	.504	97.0	<.001	
	Mean age (years)	≥65.0	−	−	−	−	–
		<65.0	−0.07 (−0.25 to 0.12)	.475	94.1	<.001	
	Statin type	Atorvastatin	0.00 (−0.19 to 0.20)	.989	67.1	.081	<.001
		Cerivastatin	0.10 (−0.04 to 0.24)	.162	−	−	
		Fluvastatin	0.00 (-0.13 to 0.13)	1.000	−	−	
		Pravastatin	−0.50 (−0.59 to −0.41)	<.001	−	−	
		Rosuvastatin	−0.01 (−0.07 to 0.04)	.695	0.0	.879	
	Follow-up duration	≥12.0	0.03 (−0.06 to 0.13)	.505	0.0	.551	.004
		<12.0	−0.13 (−0.41 to 0.15)	.360	96.7	<.001	
	Study quality	High	0.10 (−0.06 to 0.26)	.208	−	−	.013
		Low	−0.09 (−0.30 to 0.11)	.366	94.7	<.001	
Urinary protein excretion	Year of publication	Before 2010	−1.06 (−1.77 to −0.36)	.003	97.8	<.001	<.001
		2010 or after	0.05 (−0.15 to 0.24)	.621	88.7	<.001	
	Country	Asia	−0.44 (−0.86 to −0.01)	.044	90.5	<.001	.003
		Other	−0.66 (−1.15 to −0.16)	.009	98.4	<.001	
	Sample size	≥100	0.08 (−0.23 to 0.38)	.612	90.3	<.001	<.001
		<100	−0.90 (−1.46 to −0.34)	.002	98.1	<.001	
	Mean age (years)	≥65.0	−0.08 (−0.29 to 0.13)	.460	−	−	.479
		<65.0	−0.65 (−1.05 to −0.24)	.002	98.0	<.001	
	Statin type	Atorvastatin	−0.78 (−1.81 to 0.25)	.137	97.4	<.001	<.001
		Fluvastatin	−1.79 (−6.10 to 2.52)	.416	99.5	<.001	
		Pitavastatin	−1.00 (−1.33 to −0.67)	<.001	–	–	
		Pravastatin	−0.16 (−0.41 to 0.08)	.187	84.9	.010	
		Rosuvastatin	0.02 (−0.07 to 0.11)	.666	2.7	.311	
		Simvastatin	0.08 (−0.41 to 0.57)	.747	−	−	
	Follow-up duration	≥12.0	−0.85 (−1.37 to −0.33)	.001	98.5	<.001	.039
		<12.0	−0.20 (−0.78 to 0.37)	.489	95.6	<.001	
	Study quality	High	−0.39 (−0.95 to 0.18)	.182	94.6	<.001	.006
		Low	−0.69 (−1.24 to −0.15)	.012	98.3	<.001	

### Urinary albumin excretion

**Figure 3.**
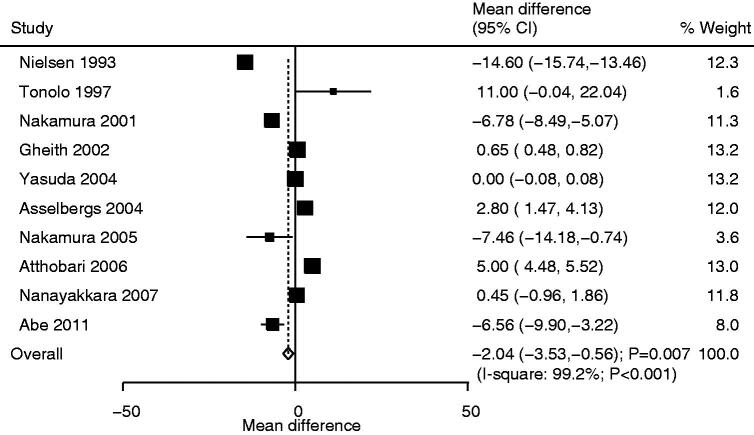
Comparison of urinary albumin excretion rate change between statin and control groups.

Ten studies reported the effect of statins on urinary albumin excretion and the pooled results suggested that the use of statins was associated with lower urinary albumin excretion than that in the control group (WMD: −2.04; 95%CI: −3.53 to −0.56; *p* = .007; Figure 3). Moreover, there was significant heterogeneity in urinary albumin excretion across the included trials (*I*^2^*^ ^*= 99.2%; *p* < .001). The conclusion was not robust when individual studies were excluded one by one (Supplemental 2). Although significant differences between statin and control groups were observed in most subgroups, we noted that statins had no significant effect on urinary albumin excretion for pooled studies conducted in Asia or other countries, sample size ≥100, mean age of patients <65.0 years, and fluvastatin or simvastatin use. Conversely, we noted that the use of statins was associated with high urinary albumin excretion for pravastatin use, follow-up duration ≥12.0 months, and high-quality studies (Table 2). No significant publication bias was observed for urinary albumin excretion (*p* value for Egger: .695; *p* value for Begg: .858; Supplemental 3).

### Creatinine clearance

**Figure 4.**
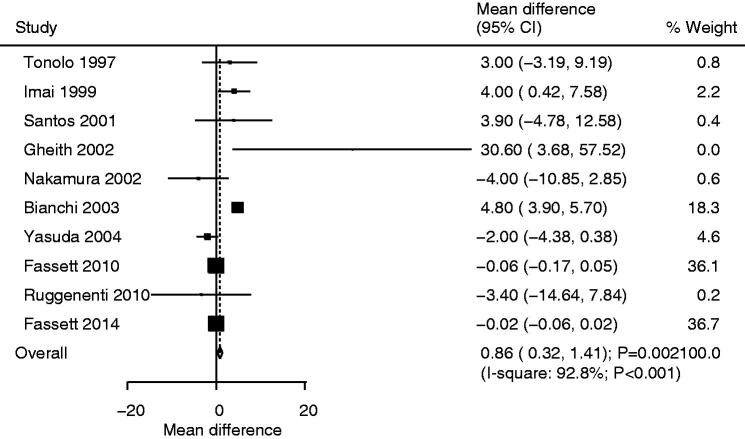
Comparison of creatinine clearance change between statin and control groups.

Ten studies reported the effects of statin use on creatinine clearance. We noted that statin use was associated with higher creatinine clearance compared with that in the control (WMD: 0.86; 95%CI: 0.32–1.41; *p* = .002; Figure 4), and significant heterogeneity was seen among the included trials (*I*^2^*^ ^*=92.8%; *p* < .001). Sensitivity analysis indicated that the pooled conclusion was not stable after the sequential exclusion of individual studies (Supplemental 2). Subgroup analysis revealed that the use of statins was associated with higher creatinine levels for pooled studies conducted in other countries, mean age <65.0 years, the use of atorvastatin, and follow-up duration ≥12.0 months (Table 2). There was no significant publication bias for creatinine clearance (*p* value for Egger: .269; *p* value for Begg: .858; Supplemental 3).

### Serum creatinine

**Figure 5.**
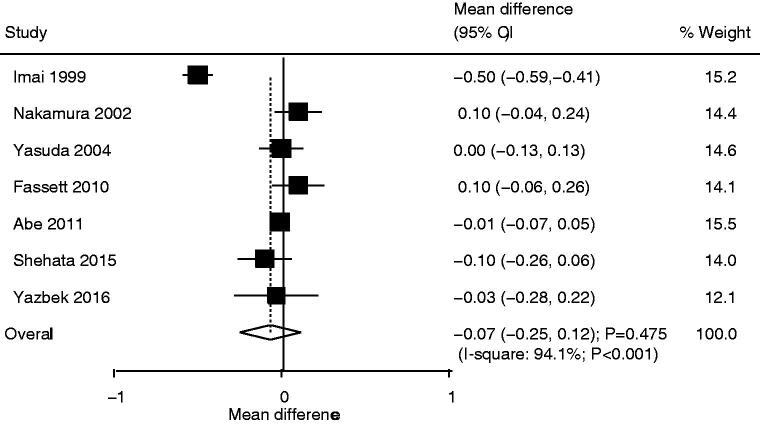
Comparison of serum creatinine change between statin and control groups.

Seven studies reported the effect of statin use on serum creatinine and the pooled result suggested no significant difference in serum creatinine levels between statin and control groups (WMD: −0.07; 95%CI: −0.25 to 0.12; *p* = .475; Figure 5). Moreover, there was significant heterogeneity among the included studies (*I*^2^*^ ^*=94.1%; *p* < .001). The pooled conclusion was robust and not altered by the sequential exclusion of individual studies (Supplemental 2). Subgroup analysis revealed that the use of pravastatin was associated with lower serum creatinine levels (Table 2). No significant publication bias for serum creatinine was observed (*p* value for Egger: .876; *p* value for Begg: .548; Supplemental 3).

### Urinary protein excretion

**Figure 6.**
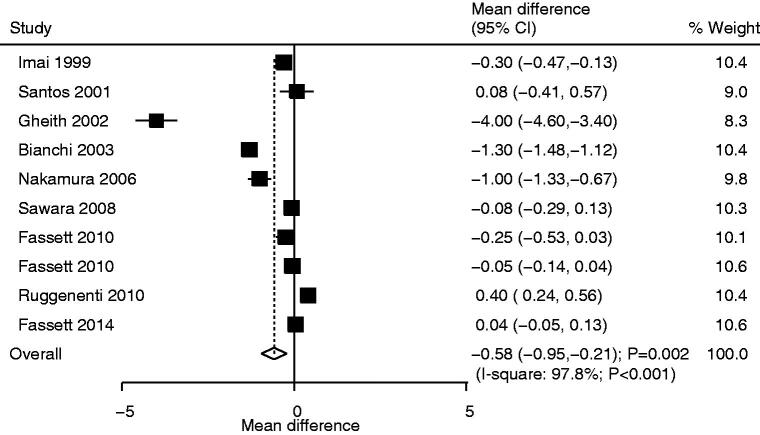
Comparison of urinary protein excretion between statin and control groups.

Ten studies reported the effect of statins on urinary protein excretion. Statin use was associated with lower urinary protein excretion (WMD: −0.58; 95%CI: −0.95 to −0.21; *p* = .002; Figure 6); moreover, we observed a significant heterogeneity for urinary protein excretion (*I*^2^*^ ^*=97.8%; *p* <.001). Sensitivity analysis indicated that the use of statins was not associated with urinary protein excretion after excluding the study conducted by Gheith et al. [31], which specifically included patients with persistent idiopathic nephrotic syndrome, and most of the patients had focal segmental glomerulosclerosis (Supplemental 2). Subgroup analysis indicated that statin use was associated with lower urinary protein excretion for pooled studies before 2010, studies conducted in Asia or other countries, sample size <100, mean age <65.0 years, the use of pitavastatin, follow-up duration ≥12.0 months, and low-quality studies (Table 2). There was no significant publication bias for urinary protein excretion (*p* value for Egger: .094; *p* value for Begg: .107; Supplemental 3).

## Discussion

This study assessed the effects of statins on renal function in patients with CKD using a meta-analytic approach. A total of 33 RCTs and 37,391 patients with CKD were identified in our study, and patient characteristics were varied. The findings of this study indicated that patients with CKD treated with statins could experience an improvement in urinary albumin excretion, creatinine clearance, and urinary protein excretion. However, statin therapy had no significant effect on eGFR and serum creatinine levels. Sensitivity analysis revealed that statin therapy might play a protective role on eGFR as compared with the control. Results of subgroup analyses suggested that the treatment efficacy of statins and control could be affected by the year of publication, country, sample size, mean age, statin type, follow-up duration, and study quality.

In a review of previous meta-analyses, Palmer et al. found that statins produce significant benefits on mortality and cardiovascular events in patients with early-stage CKD, but had no significant effects in patients on dialysis or after kidney transplantation. Moreover, they reported no significant difference between the use of statins and placebo or no treatment on eGFR [59]. However, this study reported the eGFR based on data at the end of the study, and the baseline eGFR between statin and placebo groups was not consistent. Furthermore, Shen et al. conducted a meta-analysis of 14 studies and found that the mean changes in albuminuria and urinary excretion rates in the statin group were greater than those in the placebo group, whereas no significant differences were seen between statins and placebo in terms of changes in eGFR, serum creatinine levels, or blood urea nitrogen levels [60]. However, this study included patients with diabetic nephropathy. In addition, the results of this study are consistent with those of an important meta-analysis in that participants with CKD did not require dialysis, and the effect of statins on eGFR changes was clear compared to that of the control group [61], which suggested that statin therapy may slow CKD progression by ameliorating the eGFR in patients with CKD. However, the evidence of progression to end-stage kidney disease relies on data from the SHARP Study 2010 alone, and the treatment effects of statins on this outcome remain uncertain despite the report of more than 2000 events [62]. Su et al. conducted a meta-analysis of 57 RCTs and found that statins had no significant effect on the risk of kidney failure; nonetheless, statin use could improve the decline of eGFR and proteinuria [63]. However, most studies did not involve patients with CKD. Yan et al. included six RCTs and investigated the role of high-intensity statin therapy in patients with CKD. They pointed out that patients with CKD treated with high-intensity statin therapy had a significantly reduced risk of stroke, although high-intensity statin therapy was not associated with all-cause mortality, myocardial infarction, heart failure, and renal protection [64]. Sanguankeo et al. conducted a meta-analysis of 10 RCTs and found that statins significantly improved eGFR and the beneficial effect was mainly observed for high-intensity statins [65]. However, these studies did not provide comprehensive results regarding renal function, and several published articles were not included in the meta-analysis. Therefore, the current meta-analysis was conducted to clarify the treatment efficacy of statins on renal function in patients with CKD.

The findings of this analysis were not unexpected as statins have been shown to demonstrate pleiotropic effects [15]. Statins, independent of their cholesterol-lowering effect, could ameliorate endothelial function and reduce inflammatory and fibrogenic processes in the renal interstitium [14], thereby improving renal function. However, we could not find any improvement in eGFR and serum creatinine levels. Trials investigating the effect of statins on kidney function and protein excretion yielded controversial results, with some confirming the renoprotective effect and proteinuria reduction [12] and others showing no effect [66]. In this study, we did not find a significant effect of statins on serum creatinine, although statin use was associated with a high level of urinary albumin excretion compared with that in the control group. However, this result was mainly based on a study conducted by Asselbergs et al. [36], which needed further large-scale RCT verification.

Subgroup analyses found that the beneficial effect of statins was mainly detected for studies published before 2010; studies conducted in other countries; sample size < 100; mean age of patients ≥65.0 years; the use of atorvastatin, pitavastatin, or pravastatin; or studies with low quality. The statistical power, type I error in individual trials, and quality of the included studies could explain the above results. Moreover, the baseline eGFR in elderly patients was higher than that in younger patients from several included trials, and the beneficial effects of statins might have been focused on patients with mild CKD [26,43,50]. Additionally, these results could guide further direction for the accurate evaluation of the effects of statins on renal function in patients with CKD.

This study had several limitations. First, in this review, studies that were unpublished or published in a language other than English were not included; this might have led to publication bias. Second, the heterogeneity across included studies was high and not fully interpreted through sensitivity and subgroup analyses. Third, analysis based on data from the study level and individual patient data were not available, and the detailed analyses stratified by patient characteristics were restricted. Finally, this study was not registered, and the transparency of this study was restricted.

## Conclusion

We found that patients with CKD treated with statins could experience renal function improvement by lowering the urinary albumin and protein excretions or by increasing creatinine clearance, especially with the use of atorvastatin, pitavastatin, or pravastatin. Further large-scale RCTs should be conducted to assess the long-term effects of statins on renal outcomes in patients with CKD.

## Supplementary Material

Supplemental Material

Supplemental Material

Supplemental Material

## Data Availability

The authors confirm that the data supporting the findings of this study are available within the article.

## Geolocation information

Tianjin Medical University; Tianjin; P.R. China.
