# Supplementary material for: Efficacy of statins on renal function in patients with chronic kidney disease: a systematic review and meta-analysis
Source: Ren Fail. 2021 Apr 29;43(1):718–28. doi: 10.1080/0886022X.2021.1915799 (PMC8901279; doi:10.1080/0886022X.2021.1915799)
Supplement: Supplemental Material [file IRNF_A_1915799_SM6197.pdf]

Supplementary 1

Search strategy in PubMed

1. Hydroxymethylglutaryl-CoA Reductase Inhibitors.mp. or exp Hydroxymethylglutaryl-CoA Reductase Inhibitors/

2. simvastatin.mp. or exp Simvastatin/

3. atorvastatin.mp.

4. fluvastatin.mp.

5. pravastatin.mp. or exp Pravastatin/

6. pitavastatin.mp.

7. lovastatin.mp. or exp Lovastatin/

8. hmg coa reductase inhibitor*.mp.

9. hydroxymethylglutaryl coenzyme a reductase inhibitor*.mp.

10. hmg co a reductase inhibitor*.mp.

11. mevinolin.mp.

12. pravachol.mp.

13. lipitor.mp.

14. zocor.mp.

15. mevacor.mp.

16. lescol.mp.

17. statin*.mp.

18. 1 or 2 or 3 or 4 or 5 or 6 or 7 or 8 or 9 or 10 or 11 or 12 or 13 or 14 or 15 or 16 or 17

19. renal insufficiency, chronic.mp. or exp Renal Insufficiency, Chronic/

20. exp Kidney Failure, Chronic/ or kidney failure, chronic.mp.

21. uremia.mp. or exp Uremia/

22. CKD.mp.

23. CRD.mp.

24. CRF.mp. [mp=title, abstract, original title, name of substance word, subject heading word, keyword heading word, protocol supplementary concept word, rare disease supplementary concept word, unique identifier]

25. CKF.mp. [mp=title, abstract, original title, name of substance word, subject heading word, keyword heading word, protocol supplementary concept word, rare disease supplementary concept word, unique identifier]

26. chronic renal.mp.

27. chronic kidney.mp.

28. uremi*.mp.

29. 19 or 20 or 21 or 22 or 23 or 24 or 25 or 26 or 27 or 28

30. 18 and 29

Search strategy in EmBase

(('hydroxymethylglutaryl coa reductase inhibitors'/exp or 'hydroxymethylglutaryl coa reductase inhibitors' and [embase]/lim) or ('simvastatin'/exp or 'simvastatin' and [embase]/lim) or ('atorvastatin'/exp or 'atorvastatin' and [embase]/lim) or ('fluvastatin'/exp or 'fluvastatin' and [embase]/lim) or ('pravastatin'/exp or 'pravastatin' and [embase]/lim) or ('pitavastatin'/exp or 'pitavastatin' and [embase]/lim) or ('lovastatin'/exp or 'lovastatin' and [embase]/lim) or ('hmg coa reductase inhibitor' and [embase]/lim) or ('hydroxymethylglutaryl coenzyme a reductase inhibitor' and

[embase]/lim) or ('hmg co a reductase inhibitor' and [embase]/lim) or ('mevinolin'/exp or 'mevinolin' and [embase]/lim) or (pravachol and [embase]/lim) or (lipitor and [embase]/lim) or (zocor and [embase]/lim) or (mevacor and [embase]/lim) or (lescol and [embase]/lim) or (statin* and [embase]/lim)) and (('renal insufficiency chronic'/exp or 'renal insufficiency chronic' and [embase]/lim) or ('kidney failure chronic'/exp or 'kidney failure chronic' and [embase]/lim) or ('uremia'/exp or 'uremia' and [embase]/lim) or (ckd and [embase]/lim) or (crd and [embase]/lim) or (crf and [embase]/lim) or (ckf and [embase]/lim) or ('chronic renal' and [embase]/lim) or ('chronic kidney' and [embase]/lim) or (uremi* and [embase]/lim))

Search strategy in Cochrane

#1 MeSH descriptor: [Hydroxymethylglutaryl-CoA Reductase Inhibitors] explode all trees

#2 MeSH descriptor: [Simvastatin] explode all trees

#3 MeSH descriptor: [Pravastatin] explode all trees

#4 MeSH descriptor: [Lovastatin] explode all trees

#5 atorvastatin

#6 fluvastatin

#7 pitavastatin

#8 hydroxymethylglutaryl-coa reductase inhibitor*

#9 hmg coa reductase inhibitor*

#10 hydroxymethylglutaryl coenzyme a reductase inhibitor*

#11 hmg co a reductase inhibitor*

#12 simvastatin

#13 lovastatin

#14 Pravastatin

#15 mevinolin

#16 pravachol

#17 lipitor

#18 zocor

#19 mevacor

#20 lescol

#21 statin*

#22 #1 or #2 or #3 or #4 or #5 or #6 or #7 or #8 or #9 or #10 or #11 or #12 or #13 or #14 or #15 or #16 or #17 or #18 or #19 or #20 or #21

#23 MeSH descriptor: [Renal Insufficiency, Chronic] explode all trees

#24 MeSH descriptor: [Kidney Failure, Chronic] explode all trees

#25 MeSH descriptor: [Renal Dialysis] explode all trees

#26 MeSH descriptor: [Uremia] explode all trees

#27 CKD

#28 CRD

#29 CRF

#30 CKF

#31 chronic renal

#32 chronic kidney

#33 uremi*

#34 #23 or #24 or #25 or #26 or #27 or #28 or #29 or #30 or #31 or #32 or #33

#35 #22 and #34
