# Supplementary material for: Efficacy of statins on renal function in patients with chronic kidney disease: a systematic review and meta-analysis
Source: Ren Fail. 2021 Apr 29;43(1):718–28. doi: 10.1080/0886022X.2021.1915799 (PMC8901279; doi:10.1080/0886022X.2021.1915799)
Supplement: Supplemental Material [file IRNF_A_1915799_SM6187.pdf]

Supplementary 2

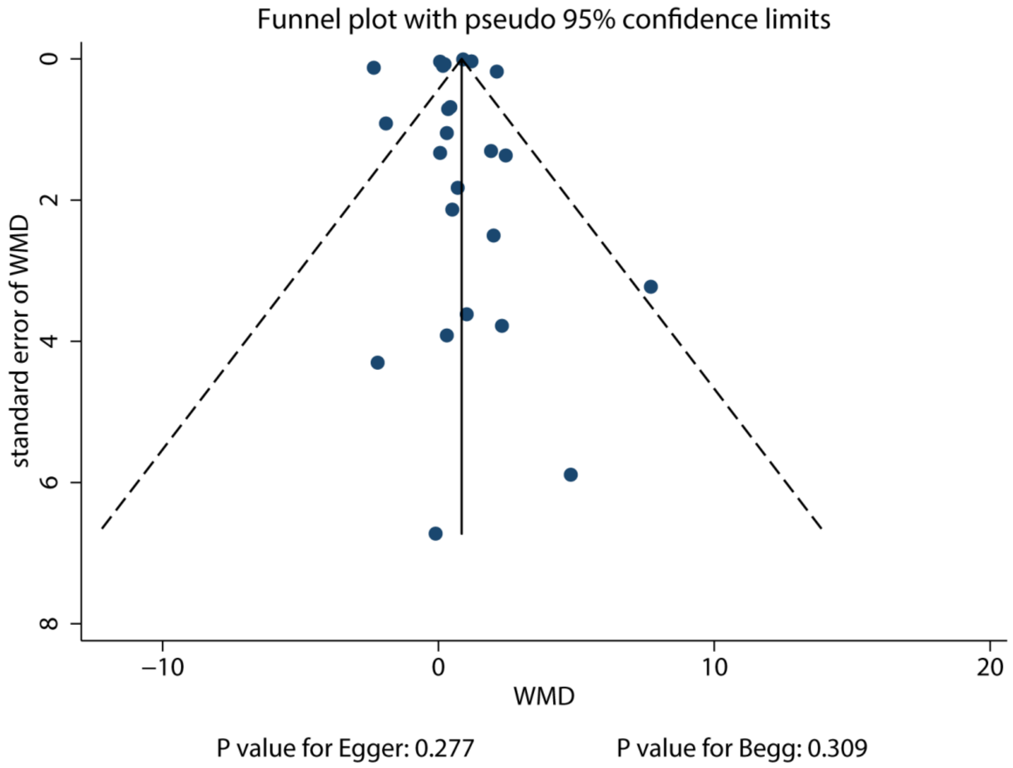

Figure S1. Funnel plot for eGFR

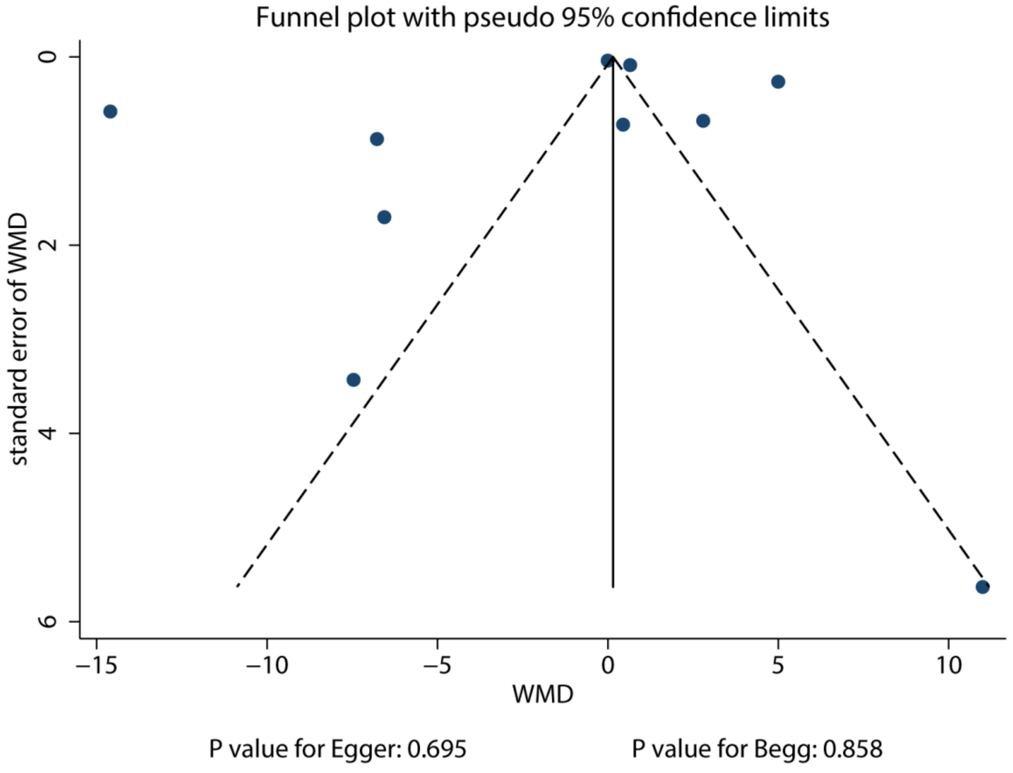

Figure S2. Funnel plot for urinary albumin excretion

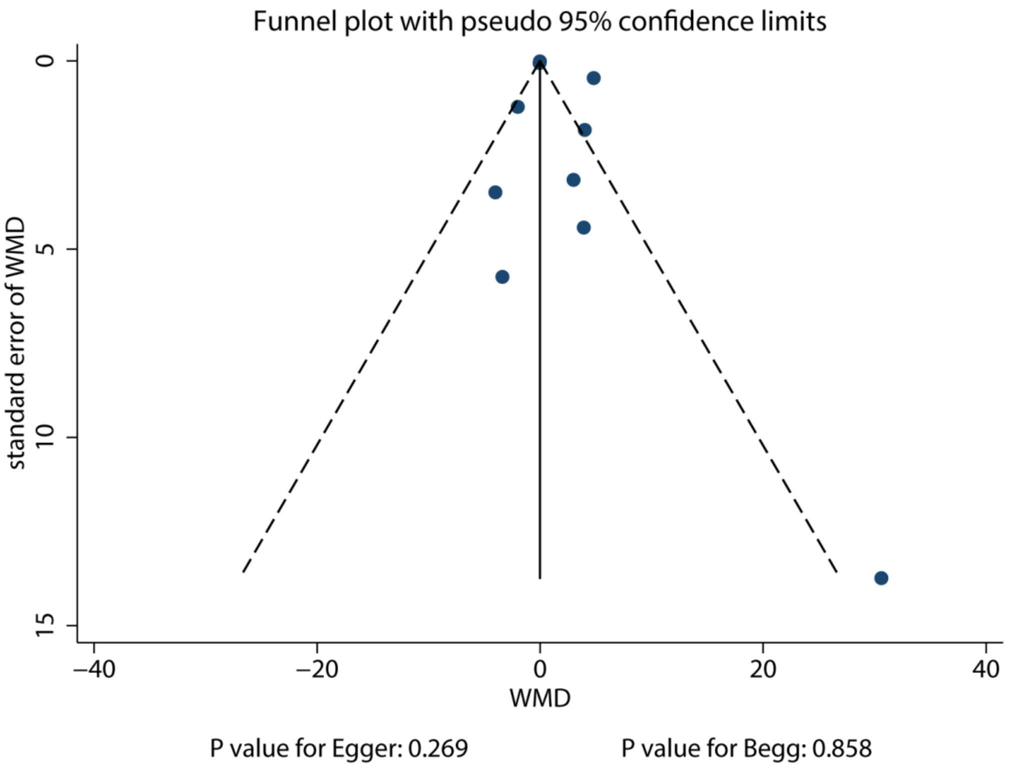

Figure S3. Funnel plot for creatinine clearance

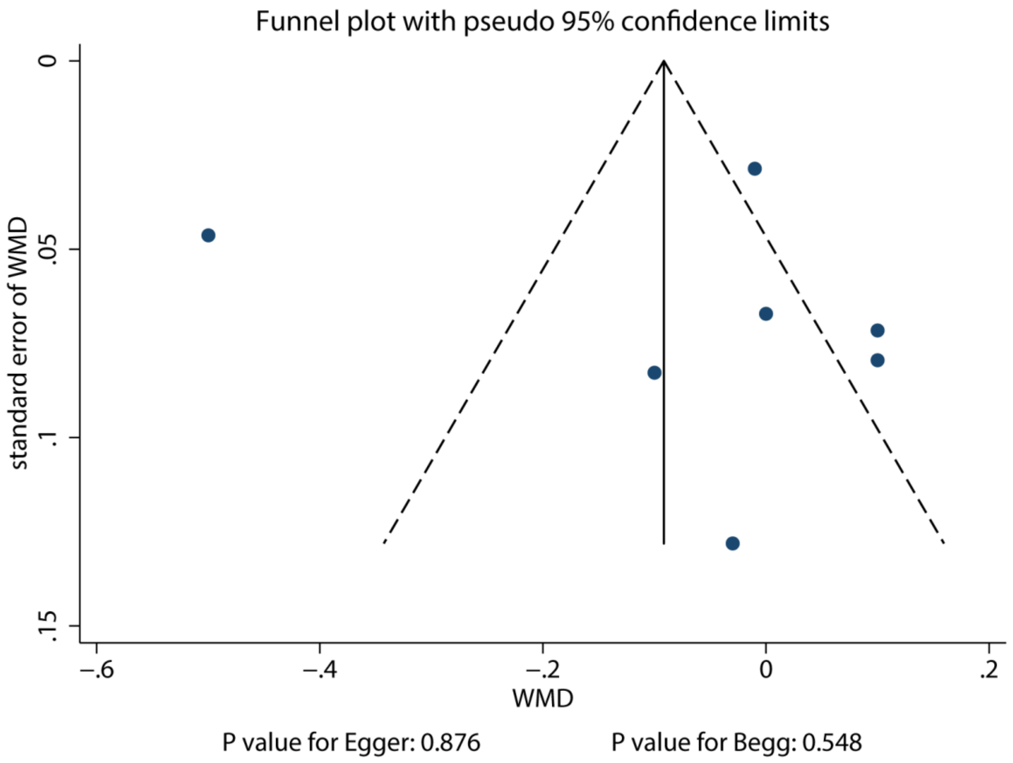

Figure S4. Funnel plot for serum creatinine

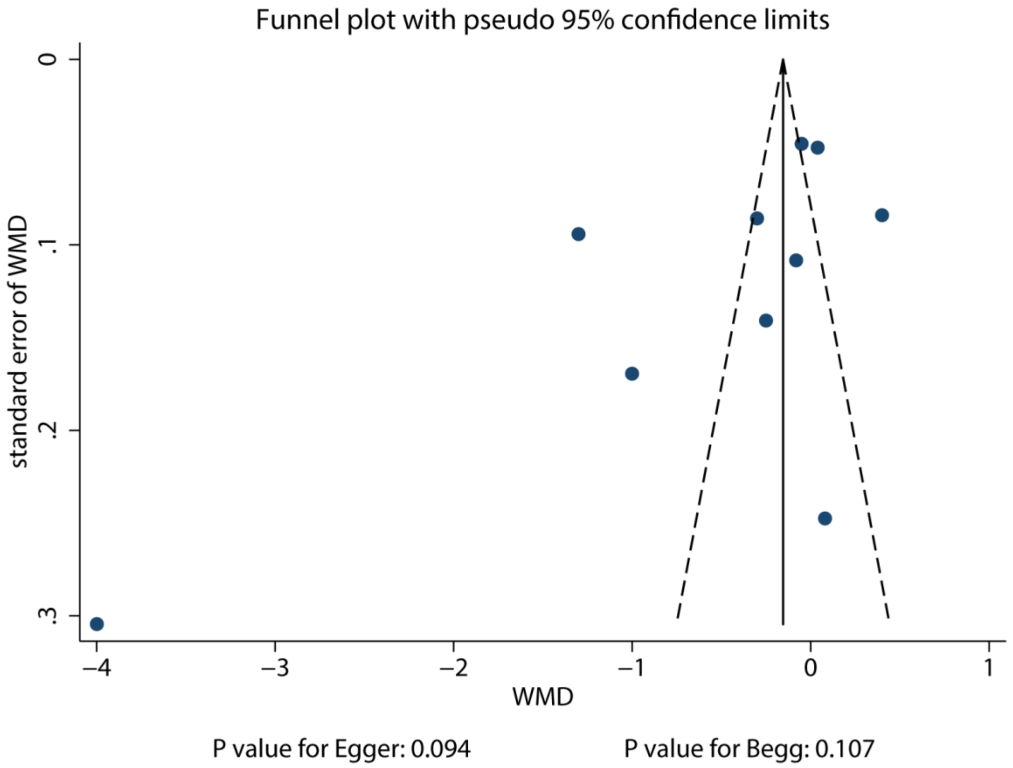

Figure S5. Funnel plot for urinary protein excretion
